# HbA_1c_ levels and breast cancer prognosis in women without diabetes

**DOI:** 10.1186/s12885-025-14121-z

**Published:** 2025-04-28

**Authors:** Jonas Busk Holm, Jens Meldgaard Bruun, Peer Christiansen, Reimar Wernich Thomsen, Jan Frystyk, Deirdre Cronin-Fenton, Signe Borgquist

**Affiliations:** 1 Department of Clinical Medicine, Aarhus University, Aarhus, Denmark; 2 Department of Oncology, Aarhus University Hospital, Aarhus, Denmark; 3 Steno Diabetes Center Aarhus, Aarhus University Hospital, Aarhus, Denmark; 4 Department of Plastic and Breast Surgery, Aarhus University Hospital, Aarhus, Denmark; 5 Department of Clinical Epidemiology, Aarhus University Hospital, Aarhus, Denmark; 6 Department of Endocrinology, Odense University Hospital, Odense, Denmark; 7 Faculty of Health Sciences, University of Southern Denmark, Odense, Denmark

**Keywords:** Breast cancer, HbA_1c_, Metabolism, Diabetes, Prognosis

## Abstract

**Background:**

Diabetes is associated with impaired breast cancer prognosis; however, the effectiveness of glycosylated hemoglobin (HbA_1c_) as a prognostic biomarker in breast cancer remains uncertain, especially for patients without diabetes. We aimed to determine whether elevated HbA_1c_ is associated with a worse prognosis in breast cancer patients without known diabetes.

**Methods:**

The study population comprised women with primary invasive stage I-III breast cancer between 2010 and 2020 surgically treated at Aarhus University Hospital, Denmark, without a diabetes diagnosis at baseline. We assessed HbA_1c_ at breast cancer diagnosis as a categorical (quartiles; HbA_1c_-Q1 = 21–33 mmol/mol, HbA_1c_-Q2 = 34–36 mmol/mol, HbA_1c_-Q3 = 37–38 mmol/mol, HbA_1c_-Q4 = ≥ 39 mmol/mol) and log2-transformed continuous variable. Follow-up began at the date of primary breast cancer surgery and continued until the first occurrence of either a new breast cancer event (loco-regional or distant recurrence, or contralateral breast cancer), new primary cancer other than breast cancer, death, emigration, or end-of-follow-up (November 15th, 2021). Cox regression models estimated crude and adjusted hazard ratios and associated 95% confidence intervals (95% CIs) of a new breast cancer event and all-cause mortality, adjusting for patient characteristics based on a directed acyclic graph. The lowest HbA_1c_ quartile (HbA_1c_-Q1) was used as reference.

**Results:**

In total, 2514 women (median age 62 years) were included. During median 5.6 years follow-up for new breast cancer events, 230 (9.1%) events occurred. An escalating risk of new breast cancer events was observed with increasing HbA_1c_ quartiles (adjusted hazard ratios, HbA_1c_-Q2: 1.09 [95% CI = 0.75–1.60]; HbA_1c_-Q3: 1.35 [95% CI = 0.88–2.07]; HbA_1c_-Q4: 1.69 [95% CI = 1.13–2.54]) compared to HbA_1c_-Q1. During median 6.0 years follow-up for all-cause mortality, 267 deaths (10.6%) occurred. No apparent association was evident between increasing HbA_1c_ quartiles and all-cause mortality (adjusted hazard ratios, HbA_1c_-Q2: 0.75 [95% CI = 0.52–1.07]; HbA_1c_-Q3: 0.82 [95% CI = 0.55–1.21]; HbA_1c_-Q4: 1.06 [95% CI = 0.74–1.53]). Similarly, a log2(HbA_1c_) increase was associated with an increased risk of new breast cancer events, but not all-cause mortality.

**Conclusions:**

For women with primary breast cancer and no known diagnosis of diabetes, higher levels of HbA_1c_ were associated with an increased risk of new breast cancer events, but not all-cause mortality. HbA_1c_ may serve as a prognostic metabolic biomarker for breast cancer patients without diabetes.

**Supplementary Information:**

The online version contains supplementary material available at 10.1186/s12885-025-14121-z.

## Introduction

Approximately 2.3 million individuals receive a breast cancer (BC) diagnosis worldwide each year [1]. Among females, BC represents one in four incident cancers and one in six cancer-related fatalities [1]. In 2021, an estimated 537 million adults worldwide had diabetes, and this number is expected to increase to 643 million by 2030 [2]. Similarly, the global obesity (Body Mass Index (BMI)$\:\ge\:$30 kg/m^2^) prevalence has more than doubled since 1990 [3]. Nearly 50% of adults are expected to have obesity by 2030 in the United States [4]. Obesity and diabetes are metabolic disorders associated with increased risk and inferior prognosis of BC [5–7]. Hyperglycemia is seen in both disorders and has been suggested as one of the mechanisms contributing to the associations with BC, as hyperglycemia may support tumor progression, for instance, through stimulation of cancer cell growth [7–12]. Continuous hyperglycemia translates into higher levels of glycosylated hemoglobin (HbA_1c_) [13]. However, the correlation between HbA_1c_ levels and BC prognosis, especially in BC patients without pre-diagnosed diabetes at the date of BC diagnosis, is ambiguous [14–21]. Earlier studies of HbA_1c_ and BC prognosis have produced contradictory findings. Some suggested an association between higher HbA_1c_ levels and poorer BC outcomes [14–16], while others found no relationship [17–21]. Most studies included patients with pre-existing diabetes at baseline and used HbA_1c_ above diabetes cutoff (HbA_1c_$\:\ge\:$48 mmol/mol [22]) as exposure [14, 15, 17–20], which does not provide a complete understanding of the role that pre-diabetic HbA_1c_ elevations play. A few of the studies counted participants without BC at baseline [16, 21]. No long-term prognostic study has concentrated solely on BC patients without diabetes at the time of their BC diagnosis, and the association between HbA_1c_ and the risk of subsequent BC events, such as recurrence or contralateral BC, has not been thoroughly examined.

We hypothesized that high HbA_1c_ is associated with inferior prognosis in BC patients without diabetes. We examined the association between HbA_1c_ levels at BC diagnosis and risk of new BC events and all-cause mortality in BC patients without diabetes.

## Materials and methods

### Data sources

The cohort has been described in detail previously [23]. We merged all data through a unique identification number assigned to all Danish residents at birth or immigration. Danish BC patients are registered in the Danish Breast Cancer Group (DBCG) database [24]. Patient, tumor, and treatment characteristics came from the DBCG database and medical records [23]. Diabetes status was collected from the Danish Adult Diabetes Registry [25]. BMI data were sourced from the Danish Anaesthesia Database [26] and medical records. We received information on emigration and comorbidities from the Civil Registration System [27] and National Patient Registry [28] included in “The Danish Clinical Quality Program - National Clinical Registries” [29]. Outcome data were gathered through a systematic review of medical records, based on a prespecified codebook [23].

### Study population

**Fig. 1:**
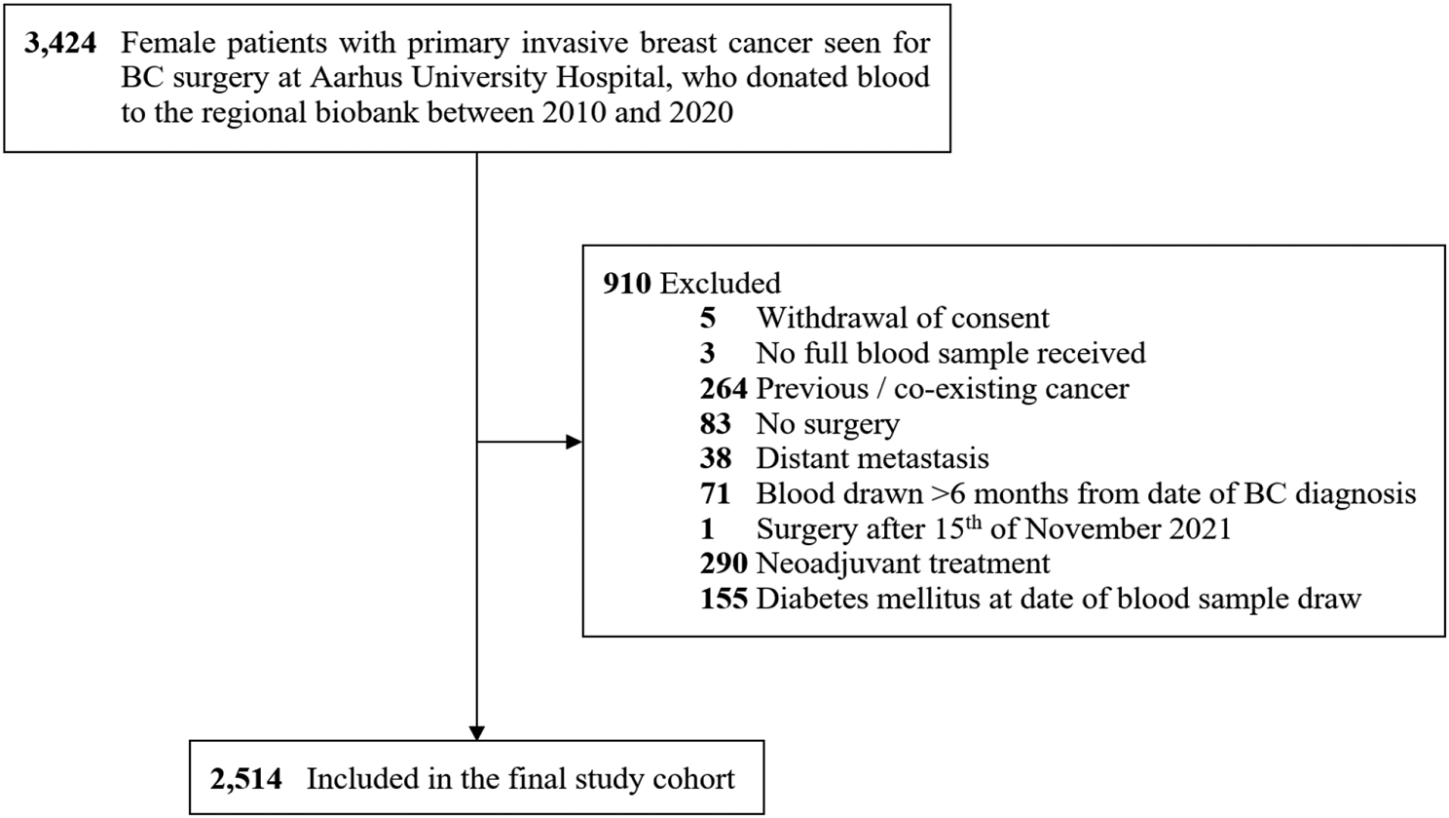
Flowchart of the study cohort. After exclusion, 2514 women with surgically treated incident stage I-III BC between 2010 and 2020 who donated blood to the regional biobank were included in the final study cohort. The women did not have a diabetes diagnosis at the date of the blood draw. This figure was modified from a previously published figure on the same cohort by Holm et al. [23]. Abbreviations: BC, Breast cancer

The study population comprised women with newly diagnosed stage I–III BC at Aarhus University Hospital, Denmark, between 2010 and 2020 [23]. These patients were referred for BC surgery at the hospital and asked to contribute blood samples to the Danish Cancer Biobank [30]. Blood was drawn a median of seven days after the primary invasive BC diagnosis (IQR 6–11 days) [23]. The final study cohort included 2514 surgically treated stage I-III BC patients without diabetes at the date of the blood sample draw (Fig. 1). The Danish National Committee on Health Research Ethics approved this study (no. 1-10-72-192-20). Informed consent was obtained from all included participants.

### HbA_1c_ analyses

Blood samples were stored at -80^o^C in the Danish Cancer Biobank [23]. HbA_1c_ levels were assessed with Sebia Capillarys 3 Tera TLA instruments [31] and reported in mmol/mol in line with the recommendations of the International Federation of Clinical Chemistry and Laboratory Medicine [32].

### Definitions of analytic variables

#### Main exposure variable

*HbA*_*1c*_*levels at BC diagnosis*. HbA_1c_ was assessed as a categorical variable and as a log2-transformed continuous variable. For the categorical analyses, HbA_1c_ was divided into quartiles based on the final study cohort (HbA_1c_-Q1 (reference) = 21–33 mmol/mol; HbA_1c_-Q2 = 34–36 mmol/mol; HbA_1c_-Q3 = 37–38 mmol/mol; HbA_1c_-Q4=$\:\ge\:$39 mmol/mol), and according to standard threshold for diagnosis of diabetes (HbA_1c_$\:\ge\:$48 mmol/mol) from the American Diabetes Association and the International Expert Committee [22, 33]. We assessed HbA_1c_ levels in quartiles, as diabetes cut-off points for HbA_1c_ may not be adequate when examining the role of HbA_1c_ in BC. For BMI-stratified analyses, we recalculated quartiles within each BMI group.

#### Covariates

*Patient characteristics.* Diabetes status refers to whether the patient was registered with diabetes in the Danish Adult Diabetes Registry [25] at the date of the blood sample draw. BMI categorization followed the World Health Organization classification [34]: underweight (BMI < 18.5 kg/m^2^), normal weight (18.5 ≤ BMI < 25 kg/m^2^), overweight (25 ≤ BMI < 30 kg/m^2^), and obesity (BMI ≥ 30 kg/m^2^). We used the closest registered BMI measurement from the blood sample date [23]. Menopausal status and age (following DBCG guidelines [24]) referred to the time of BC diagnosis. Comorbidities were assessed as Charlson Comorbidity Index score (0, 1–2 (mild), & $\:\ge\:$3 (moderate/severe)), incorporating comorbidities registered in the National Patient Registry up to 10 years before BC diagnosis [35].

*Tumor characteristics.* Tumor size and lymph node metastases were categorized according to the American Joint Committee on Cancer Staging 8th edition [36]. We classified tumors as ER (estrogen receptor)-negative if tumor cells showed no expression of ER, or ER-positive if $\:\ge\:$1% expressed ER. Human Epidermal Growth Factor Receptor 2 (HER2) expression was defined as positive or negative through immunohistochemistry and Fluorescence In Situ Hybridization (FISH)-ratio according to guidelines by the American Society of Clinical Oncology [37]. Nottingham Group standardization was used for histological grade [36] and the World Health Organization’s Classification of Breast Tumors 3rd Edition for histological classification [38].

*Treatment characteristics.* Surgery type (mastectomy or lumpectomy) referred to the final primary BC surgery [23]. Endocrine therapy, chemotherapy, anti-HER2 therapy, and radiotherapy were included as intention-to-treat variables (defined by DBCG protocols).

#### Outcomes

We defined BC recurrence as recurrent invasive BC in the ipsilateral breast or ipsilateral lymph nodes (loco-regional), or outside of these locations (distant) ≥ 3 months after the last surgery date for primary BC (final primary surgery) [23]. We only included the first registered BC recurrence. Malignancy in the contralateral breast after final primary surgery was registered as contralateral BC [23]. We also registered deaths and new primary cancers other than BC, apart from non-melanoma skin cancer [23]. BC recurrence (loco-regional or distant) and contralateral BC were considered new BC events.

### Statistical analyses

Follow-up for new BC events and distant recurrence started on the final primary surgery date and continued until one of the following: invasive BC recurrence (loco-regional or distant), contralateral BC, new primary cancer other than BC, death, emigration, or last follow-up date (November 15th, 2021). In follow-up for new BC events, we treated BC recurrence (loco-regional or distant) and contralateral BC as events and censored at new primary cancer, death, emigration, or November 15th, 2021. For the Aalen-Johansen estimators, competing events were new primary cancer and death. For distant recurrence follow-up, distant BC recurrence was treated as an event, and censoring occurred at loco-regional BC recurrence, contralateral BC, new primary cancer, death, emigration, or November 15th, 2021. If an event occurred within 30 days after a censoring point, we ignored the censoring point and included the event in the analysis.

All-cause mortality follow-up began at the final primary surgery date and continued until one of the following: death, emigration, or November 15th, 2021. We treated death as an event and censored at emigration or November 15th, 2021.

Person-years, number of events, and incidence rate per 1000 person-years (with 95% confidence intervals (95% CIs)) within each HbA_1c_ quartile were calculated. Crude and multivariable-adjusted hazard ratios (HRs) with 95% CIs for new BC events, distant recurrence, and all-cause mortality were estimated using Cox models in relation to categorized HbA_1c_ and log2(HbA_1c_). The Cox models were utilized for a maximum follow-up of 10 years. Two multivariable models were run. Model 1 was adjusted for crucial confounders, as indicated by a directed acyclic graph, namely age, menopausal status, BMI, and comorbidities (Supplementary Fig. 1). Model 2 was exploratory and included tumor and treatment characteristics potentially mediating the impact of HbA_1c_ on BC prognosis, rather than qualifying as confounders. These covariates included ER status, HER2 receptor status, grade, classification, tumor size, lymph node metastases, surgery, radiotherapy, endocrine therapy, chemotherapy, and anti-HER2 therapy. This exploratory model intended to assess whether the observed associations would likely act through these factors. Only patients with complete data in the covariates were included (*N* = 2448 (model 1)/2336 (model 2)). We also conducted BMI-stratified analyses, ER-stratified analyses, and sensitivity analyses excluding patients with HbA_1c_$\:\ge\:$48 mmol/mol (diabetes threshold [22]). For distant recurrence, BMI-stratified, and ER-stratified analyses, model 2 was not presented due to an insufficient number of events [39]. Patients classified as underweight were omitted from the BMI-stratified analyses because of the low number of such patients. We provided Aalen-Johansen estimators on the cumulative incidence of new BC events, and Kaplan-Meier estimators on the cumulative incidence of all-cause mortality, according to HbA_1c_ quartiles overall and within BMI groups. All analyses were conducted using Stata version 18.

## Results

The study cohort comprised 2514 stage I–III BC patients without diabetes. The median age was 62 years (IQR 52–69), median BMI was 24.7 kg/m^2^, and 349 patients (13.9%) had a Charlson Comorbidity Index score $\:\ge\:$3 (Table 1). Patients in the highest HbA_1c_ quartile (HbA_1c_-Q4, $\:\ge\:$39 mmol/mol) were older, more likely to be postmenopausal, had higher BMI, and more comorbidity. Patients in HbA_1c_-Q4 had larger tumors, more often exhibited ductal carcinomas, and were less likely to undergo chemotherapy. The characteristics of the patients with complete data (Model 2, *N*=2336) were similar to those in the crude analyses (*N* = 2514) (Supplementary Table 1).

**Table 1:** Characteristics of 2514 women with incident stage I-III breast cancer included in the final cohort

Characteristics	Total *N* = 2,514	HbA_1c_-Q1 21–33 mmol/mol *N* = 649	HbA_1c_-Q2 34-36 mmol/mol *N* = 794	HbA_1c_-Q3 37-38 mmol/mol *N* = 485	HbA_1c_-Q4 $\:\ge\:$39 mmol/ *N* = 586
**Age**,** median (IQR)**	62 (52–69)	52 (47–64)	61 (52–68)	64 (58–71)	67 (59–73)
**Age (years)**,** categories**					
< 50	441 (17.5%)	229 (35.3%)	137 (17.3%)	42 (8.7%)	33 (5.6%)
50–59	639 (25.4%)	181 (27.9%)	228 (28.7%)	112 (23.1%)	118 (20.1%)
60–69	852 (33.9%)	156 (24.0%)	274 (34.5%)	198 (40.8%)	224 (38.2%)
$\:\ge\:$ 70	582 (23.2%)	83 (12.8%)	155 (19.5%)	133 (27.4%)	211 (36.0%)
Missing	0	0	0	0	0
**Body Mass Index (kg/m**^**2**^**)**,** median (IQR)**	24.74 (22.20-28.09)	23.51 (21.56–25.94)	24.34 (22.04–27.48)	25.06 (22.15–28.24)	27.10 (24.11–31.23)
**Body Mass Index**,** categories (kg/m**^**2**^**)**					
Underweight < 18.5	62 (2.5%)	16 (2.5%)	22 (2.8%)	15 (3.1%)	9 (1.6%)
Normal weight 18.5 $\:\le\:$ to < 25	1,229 (49.6%)	406 (63.6%)	418 (53.4%)	219 (45.6%)	186 (32.3%)
Overweight 25 ≤ to < 30	769 (31.1%)	163 (25.5%)	231 (29.5%)	171 (35.6%)	204 (35.5%)
Obesity ≥ 30	416 (16.8%)	53 (8.3%)	112 (14.3%)	75 (15.6%)	176 (30.6%)
Missing	38	11	11	5	11
**Menopausal status**					
Premenopausal	584 (23.5%)	276 (43.0%)	195 (25.0%)	64 (13.2%)	49 (8.5%)
Postmenopausal	1902 (76.5%)	366 (57.0%)	586 (75.0%)	420 (86.8%)	530 (91.5%)
Missing	28	7	13	1	7
**Charlson Comorbidity Index**					
0	341 (13.6%)	106 (16.3%)	105 (13.2%)	63 (13.0%)	67 (11.4%)
1–2 (mild)	1824 (72.6%)	471 (72.6%)	599 (75.4%)	365 (75.3%)	389 (66.4%)
$\:\ge\:$3 (moderate/severe)	349 (13.9%)	72 (11.1%)	90 (11.3%)	57 (11.8%)	130 (22.2%)
Missing	0	0	0	0	0
**Tumor size**					
0–20 mm	1782 (71.0%)	474 (73.1%)	578 (73.1%)	345 (71.1%)	385 (65.8%)
21–50 mm	675 (26.9%)	162 (25.0%)	197 (24.9%)	132 (27.2%)	184 (31.5%)
> 50 mm	52 (2.1%)	12 (1.9%)	16 (2.0%)	8 (1.6%)	16 (2.7%)
Missing	5	1	3	0	1
**Lymph node metastases**					
0	1560 (62.6%)	387 (60.2%)	496 (62.9%)	304 (63.2%)	373 (64.3%)
1–3	693 (27.8%)	196 (30.5%)	218 (27.6%)	130 (27.0%)	149 (25.7%)
4–9	167 (6.7%)	42 (6.5%)	52 (6.6%)	34 (7.1%)	39 (6.7%)
$\:\ge\:$10	73 (2.9%)	18 (2.8%)	23 (2.9%)	13 (2.7%)	19 (3.3%)
Missing	21	6	5	4	6
**Histological classification**					
Ductal	1892 (75.3%)	474 (73.0%)	593 (74.7%)	373 (76.9%)	452 (77.1%)
Lobular	309 (12.3%)	89 (13.7%)	101 (12.7%)	57 (11.8%)	62 (10.6%)
Other^a^	313 (12.5%)	86 (13.3%)	100 (12.6%)	55 (11.3%)	72 (12.3%)
Missing	0	0	0	0	0
**Histological grade**					
Not graded^b^	154 (6.2%)	43 (6.7%)	50 (6.5%)	33 (6.9%)	28 (4.9%)
Grade 1	579 (23.5%)	151 (23.6%)	185 (23.9%)	108 (22.5%)	135 (23.4%)
Grade 2	1140 (46.2%)	282 (44.1%)	350 (45.2%)	233 (48.5%)	275 (47.7%)
Grade 3	596 (24.1%)	163 (25.5%)	189 (24.4%)	106 (22.1%)	138 (24.0%)
Missing	45	10	20	5	10
**ER status (% positive cells)**					
0% (negative)	252 (10.1%)	89 (13.7%)	67 (8.5%)	45 (9.3%)	51 (8.8%)
1-100% (positive)	2249 (89.9%)	559 (86.3%)	722 (91.5%)	438 (90.7%)	530 (91.2%)
Missing	13	1	5	2	5
**HER2 status**					
Negative	2200 (89.3%)	565 (88.1%)	701 (90.2%)	423 (89.8%)	511 (89.0%)
Positive	263 (10.7%)	76 (11.9%)	76 (9.8%)	48 (10.2%)	63 (11.0%)
Missing	51	8	17	14	12
**Final primary surgery** ^c^					
Mastectomy	829 (33.2%)	219 (33.9%)	253 (32.1%)	151 (31.2%)	206 (35.4%)
Lumpectomy	1671 (66.8%)	427 (66.1%)	535 (67.9%)	333 (68.8%)	376 (64.6%)
Missing	14	3	6	1	4
**Adjuvant radiotherapy** ^d^					
No	488 (20.0%)	121 (19.1%)	152 (19.7%)	90 (19.1%)	125 (22.2%)
Yes	1950 (80.0%)	512 (80.9%)	619 (80.3%)	380 (80.9%)	439 (77.8%)
Missing	76	16	23	15	22
**Endocrine therapy** ^d^					
No	475 (19.5%)	146 (23.1%)	133 (17.3%)	92 (19.6%)	104 (18.4%)
Yes	1963 (80.5%)	487 (76.9%)	638 (82.7%)	378 (80.4%)	460 (81.6%)
Missing	76	16	23	15	22
**Anti-HER2 therapy** ^d^					
No	2179 (89.2%)	559 (88.0%)	696 (90.2%)	421 (89.8%)	503 (88.9%)
Yes	263 (10.8%)	76 (12.0%)	76 (9.8%)	48 (10.2%)	63 (11.1%)
Missing	72	14	22	16	20
**Adjuvant chemotherapy** ^d^					
No	1199 (49.2%)	235 (37.1%)	378 (49.0%)	258 (54.9%)	328 (58.2%)
Yes	1239 (50.8%)	398 (62.9%)	393 (51.0%)	212 (45.1%)	236 (41.8%)
Missing	76	16	23	15	22

a: “Other” refers to patients without registration of either invasive ductal or lobular carcinoma

b: In total, 154 patients’ tumors were not graded during the histological assessment, e.g. due to nonductal and nonlobular carcinomas were not graded for part of the study period or insufficient amount of tumor tissue for grading. We did not treat “Not graded” as a missing value in the multivariable models

c: Final primary surgery refers to the last breast surgery procedure for the primary breast cancer

d: Intention-to-treat variables based on the Danish Breast Cancer Group protocol allocation

Abbreviations: Q1, Quartile 1; IQR, Interquartile range; ER, Estrogen receptor; HER2, Human Epidermal Growth Factor Receptor 2

Among the 2514 patients with BC, we registered 230 new BC events (195 recurrences and 35 contralateral BCs) during 14,126 person-years of follow-up for new BC events (median follow-up of 5.6 years). In follow-up for all-cause mortality, 267 deaths occurred during 14,913 person-years (median follow-up of 6.0 years).

**Fig. 2:**
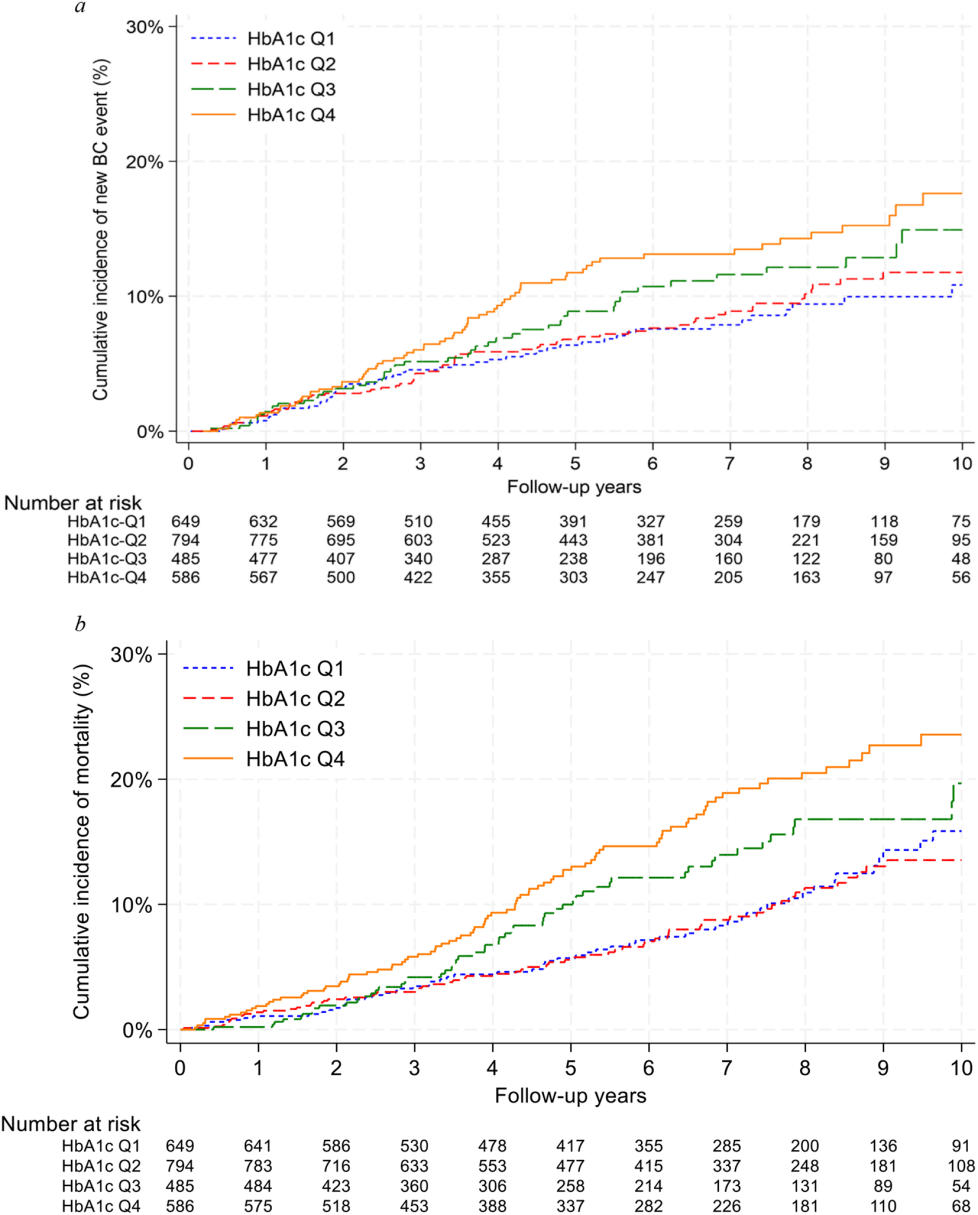
Cumulative new breast cancer event and all-cause mortality incidences across HbA_1c_ quartiles. **a**: Cumulative new breast cancer event (BC recurrence or contralateral BC) incidences across HbA_1c_ quartiles (Aalen-Johansen estimator). Competing events: new primary cancer other than BC and death. **b**: Cumulative all-cause mortality incidences across HbA_1c_ quartiles (Kaplan-Meier estimator). Abbreviations: BC, Breast cancer; HbA1c Q1, HbA_1c_ quartile 1

Figure 2 displays the cumulative new BC event and all-cause mortality incidences across HbA_1c_ quartiles.

The highest BC events and mortality incidences were observed in HbA_1c_-Q4. Table 2 provides the estimated HRs for a new BC event, distant recurrence, and all-cause mortality across HbA_1c_ quartiles. Among the 230 new BC events, the bulk occurred in HbA_1c_-Q4 (*N* = 71; 30.9%). An increased risk of a new BC event with rising HbA_1c_ quartiles was observed (Model 1, HbA_1c_-Q2: adjusted HR = 1.09, 95% CI = 0.75–1.60; HbA_1c_-Q3: adjusted HR = 1.35, 95% CI = 0.88–2.07; HbA_1c_-Q4: adjusted HR = 1.69, 95% CI = 1.13–2.54). Similarly, a higher hazard of distant recurrence was observed in HbA_1c_-Q4 compared to HbA_1c_-Q1 (Model 1, HbA_1c_-Q4, adjusted HR = 2.09 [95% CI = 1.23–3.56]). Out of the 267 recorded deaths, the majority were in HbA_1c_-Q4 (*N* = 91; 34.1%). No clearly increased mortality risk across quartiles compared to HbA_1c_-Q1 was noted (Model 1, HbA_1c_-Q2: adjusted HR = 0.75, 95% CI = 0.52–1.07; HbA_1c_-Q3: adjusted HR = 0.82, 95% CI = 0.55–1.21; HbA_1c_-Q4: adjusted HR = 1.06, 95% CI = 0.74–1.53). When considering HbA_1c_ as a continuous variable, an association between an increase in log2(HbA_1c_) and increased risk of new BC events (Model 1, adjusted HR = 1.95 [95% CI = 0.92–4.12]) and distant recurrence (Model 1, adjusted HR = 2.50 [95% CI = 1.01–6.20]) was observed.

**Table 2:** Outcome estimates according to HbA_1c_ quartiles and log2(HbA_1c_) in the 2514 breast cancer patients

	Person-years	Number of events	Incidence rate per 1000 person-years (95% CI)	Crude hazard ratio (95% CI) (*N* = 2514)	Model 1: Hazard ratio adjusted for confounders based on directed acyclic graph (95% CI)^a^ (*N* = 2448)	Model 2: Adjusted hazard ratio (95% CI)^b^ (*N* = 2336)
**New breast cancer event**						
Q1 (21–33 mmol/mol) (*N* = 649)	3791	49	12.93 (9.77–17.10)	1 [Reference]	1 [Reference]	1 [Reference]
Q2 (34–36 mmol/mol) (*N* = 794)	4547	64	14.08 (11.02–17.98)	1.09 (0.75–1.58)	1.09 (0.75–1.60)	1.24 (0.83–1.86)
Q3 (37–38 mmol/mol) (*N* = 485)	2593	46	17.74 (13.29–23.68)	1.37 (0.92–2.05)	1.35 (0.88–2.07)	1.37 (0.87–2.15)
Q4 ($\:\ge\:$39 mmol/mol) (*N*=586)	3195	71	22.22 (17.61–28.04)	1.72 (1.19–2.47)	1.69 (1.13–2.54)	1.82 (1.17–2.81)
Total (*N* = 2514)	14,126	230^c^				
Per HbA_1c_ log2 increase				2.28 (1.18–4.41)	1.95 (0.92–4.12)	1.86 (0.84–4.10)
**Distant recurrence**						
Q1	3791	27	7.12 (4.88–10.39)	1 [Reference]	1 [Reference]	NA
Q2	4547	38	8.36 (6.08–11.49)	1.18 (0.72–1.93)	1.23 (0.75–2.04)	NA
Q3	2593	26	10.03 (6.83–14.73)	1.42 (0.83–2.44)	1.53 (0.87–2.70)	NA
Q4	3195	46	14.40 (10.78–19.22)	2.04 (1.27–3.28)	2.09 (1.23–3.56)	NA
Total	14,126	137^d^				
Per HbA_1c_ log2 increase				2.93 (1.31–6.52)	2.50 (1.01–6.20)	NA
**All-cause mortality**						
Q1	3987	57	14.30 (11.03–18.53)	1 [Reference]	1 [Reference]	1 [Reference]
Q2	4787	65	13.58 (10.65–17.31)	0.95 (0.67–1.36)	0.75 (0.52–1.07)	0.81 (0.55–1.20)
Q3	2723	54	19.83 (15.19–25.89)	1.41 (0.97–2.05)	0.82 (0.55–1.21)	0.79 (0.52–1.20)
Q4	3415	91	26.65 (21.70-32.73)	1.88 (1.35–2.61)	1.06 (0.74–1.53)	1.10 (0.75–1.62)
Total	14,913	267				
Per HbA_1c_ log2 increase				2.85 (1.62–5.02)	1.05 (0.51–2.17)	0.96 (0.45–2.05)

a: Adjusted for age, menopausal status, comorbidities, and body mass index

b: Adjusted for age, menopausal status, comorbidities, body mass index, estrogen receptor status, HER2 receptor status, histological grade, tumor size, lymph node metastases, histological classification, surgery, radiotherapy, and systemic treatment (endocrine therapy, chemotherapy, and anti-HER2 therapy)

c: 195 breast cancer recurrences, 35 contralateral breast cancers

d: 83 visceral metastases and 54 bone metastases

HRs for new BC events, distant recurrence, and all-cause mortality excluding patients with HbA_1c_$\:\ge\:$48 mmol/mol are presented in Supplementary Table 2, where the increased risk of new BC events and distant recurrence in HbA_1c_-Q4 compared to HbA_1c_-Q1 is replicated– similar to Table 2’s results. In Supplementary Table 3, we present HRs for a new BC event, distant recurrence, and all-cause mortality using HbA_1c_ cut-off points according to the International Expert Committee and American Diabetes Association [22, 33]. The pre-diabetes groups (International Expert Committee: HbA_1c_=42–47 mmol/mol; American Diabetes Association: HbA_1c_=39–47 mmol/mol) had the highest risk of inferior BC prognosis, similar to results in Table 2 and Supplementary Table 2 [22, 33]. In the ER-stratified analyses, patients in HbA_1c_-Q4 had the highest risk of a new BC event regardless of ER status (Supplementary Table 4).

In Supplementary Figs. 2 and 3, we present BMI-stratified cumulative BC event and all-cause mortality incidences according to HbA_1c_ quartiles. For patients with normal weight, we observed the highest BC event and mortality incidences in HbA_1c_-Q4 compared to other quartiles. Similar results were observed in patients with obesity, but not overweight. In Supplementary Table 5, we report estimated HRs across HbA_1c_ quartiles in BMI groups. We saw a tendency of increased risk of new BC events with increasing HbA_1c_ quartiles in patients with normal weight or obesity. Increasing HbA_1c_ quartiles were not associated with mortality across BMI groups.

## Discussion

In BC patients without diabetes at baseline, we found an escalating risk of new BC events (recurrence or contralateral BC) with increasing HbA_1c_ quartiles. In the highest quartile (HbA_1c_$\:\ge\:$39 mmol/mol), patients had a 69% enhanced risk of a new BC event and twice the risk of distant recurrence compared with patients in the lowest HbA_1c_ quartile. However, elevated HbA_1c_ (HbA_1c_$\:\ge\:$39 mmol/mol) was not independently related to an increased risk of all-cause mortality.

Previous literature has reported inconsistent findings on the relationship between HbA_1c_ and BC prognosis. In patients with diabetes at BC diagnosis, Laurberg et al. (*N* = 1978) and Boursi et al. (*N* = 1382) found no association between HbA_1c_ and overall survival [18, 19]. Similarly, Tobe et al. (*N* = 98) found no association with overall or distant metastasis-free survival [17]. For patients with mixed diabetes status (i.e., patients with or without diabetes), Erickson et al. [14] reported an increased risk of all-cause mortality and slightly increased risk of a new BC event (recurrence or new primary BC) for patients with HbA_1c_ ≥ 53 mmol/mol compared to HbA_1c_ < 48 mmol/mol in a cohort of 3003 BC patients. Similarly, Chang et al. [15] found an increased risk of BC-specific and all-cause mortality in BC patients with diabetes and HbA_1c_ > 75 mmol/mol compared to patients without diabetes (*N* = 2812). Conversely, no association between HbA_1c_ and overall survival was found by Jousheghany et al. [20] in patients with unknown diabetes status at BC diagnosis (*N* = 82). Two studies explored the relationship between HbA_1c_ and BC prognosis in patients without diabetes at enrolment [16, 21]. Yoo et al. [16] found a slightly increased risk of BC-specific mortality for patients with the highest HbA_1c_-quintile (39–46 mmol/mol) compared to the lowest quintile among 589,457 patients. The number of BC-specific mortalities and the precision of the estimate were low [16]. Conversely, Joshu et al. [21] found no association between HbA_1c_ and BC-specific mortality in 336 BC patients.

Direct comparison between our results and prior studies is challenging due to differences in study design, the timing of HbA_1c_ measurement, diverse endpoints, and exposure thresholds, and the inclusion/exclusion of patients with diabetes. As in the most comprehensive study on BC patients [14], we noted an increased risk of new BC events within the highest HbA_1c_ group, though no association with mortality was found. In contrast, Erickson et al. [14] incorporated patients with self-reported diabetes, and used HbA_1c_$\:\ge\:$53 mmol/mol as the exposure threshold. Also, HbA_1c_ was measured in samples taken on average two years post-BC diagnosis [14]. Like Tobe et al. [17], Laurberg et al. [18], and Boursi et al. [19], we did not observe a link between HbA_1c_ and risk of all-cause mortality, but in contrast to our study, all three studies only included patients with diabetes. Contrary to Tobe et al. [17], we found an association between HbA_1c_ and distant recurrence risk. Comparing our results to Chang et al. [15] is problematic, since their exposure was HbA_1c_ levels in patients with diabetes, and patients without diabetes were the reference group. Yoo et al. [16] and Joshu et al. [21] included individuals without cancer at start, measuring HbA_1c_ before BC diagnosis, contrasting with our design. Furthermore, Yoo et al. [16] provided no information on the number of BC cases.

Our study is the first to report results on the association between HbA_1c_ at BC diagnosis and BC prognosis in patients without diabetes at baseline. The majority of studies on HbA_1c_ and BC prognosis have investigated HbA_1c_ synonymous with diabetes as exposure (HbA_1c_$\:\ge\:$48 mmol/mol) [14, 15, 17, 20, 22]. Erickson et al. [14] questioned whether there was a threshold of glycemic status at which the risk of poor BC prognosis significantly increases. Our study indicates that HbA_1c_ in the non-diabetic range (HbA_1c_<48 mmol/mol) is associated with inferior prognosis in BC patients. Therefore, diabetes cut-off points for HbA_1c_ might be insufficient in BC, however, HbA_1c_ in the non-diabetic range may not affect mortality risk, as we saw no association between HbA_1c_-Q4 and all-cause mortality, opposite to Erickson et al. [14].

Our observed increased risk of new BC events and distant recurrences associated with HbA_1c_-Q4 could be attributed to several factors. In BC cell lines, high glucose induced BC cell invasion through epithelial-to-mesenchymal transition [12, 40, 41], a critical element in cancer metastasis [42]. This could explain the heightened risk of distant recurrence. Moreover, high glucose may increase the proliferation of BC cells, leading to larger tumors in HbA_1c_-Q4 [12, 41]. Additionally, elevated HbA_1c_ could suggest hyperinsulinemia and systemic inflammation, as these conditions are frequently observed in patients with type 2 diabetes [2, 43, 44]. Since hyperinsulinemia and systemic inflammation are associated with poor BC outcomes, they could account for the association between increased HbA_1c_ and unfavorable BC prognosis [8, 45, 46].

Our study may have clinical implications. HbA_1c_ within the non-diabetic range could be included in the clinical evaluation of the risk of new BC events. Furthermore, as high HbA_1c_ levels were associated with an increased risk of new BC events in patients with normal weight, HbA_1c_ levels may help identify patients who are not metabolically healthy to an extent that affects cancer prognosis even if the patient has a “healthy” BMI. Also, despite a lower precision of the estimate in patients with obesity, HbA_1c_ may be a relevant prognostic marker in this group, too. It should be noted that all BMI-stratified results have low precision due to few events.

### Limitations

This study has certain limitations. Our cohort encompassed only those patients diagnosed and treated for BC at one institution, who agreed to participate [23]. We lack information on the number of patients who declined to donate blood to the biobank. Nevertheless, when comparing our cohort to the number of breast cancer patients registered in the annual reports by the DBCG [47], and seen at the BC surgery department at Aarhus University Hospital during our inclusion period, the participation rate exceeds 90%. This is our best estimate, but still, we cannot overlook potential selection issues. Additionally, there may be some misclassification at baseline among the patients included, considering the majority, but not all patients with diabetes in Denmark, are registered in the Danish Adult Diabetes Registry [48]. Furthermore, HbA_1c_ was only assessed once. Also, the blood samples were stored in the freezer for a median of 8.1 years, which could affect the reliability of the HbA1c measurements. However, according to Selvin et al., HbA_1c_ levels in long-term stored frozen whole blood samples correlate highly with measurements done before the storage of the samples [49, 50]. Lastly, the possibility of residual confounding, such as hyperinsulinemia and inflammation, cannot be excluded.

## Conclusions

In BC patients without known diabetes, elevated HbA_1c_ levels (HbA_1c_$\:\ge\:$39 mmol/mol) were associated with an increased risk of new BC events, but not with all-cause mortality. These findings imply that HbA_1c_ levels might have prognostic value for BC assessment, even in the non-diabetic range, thereby helping clinicians identify patients with a poorer BC prognosis. Consequently, these results also prompt the question of whether closer monitoring and treatment of pre-diabetic hyperglycemia could improve BC prognosis.

## Electronic supplementary material

Below is the link to the electronic supplementary material.

Supplementary Material 1

## Data Availability

The datasets generated and/or analyzed during the current study are not publicly available due individual privacy could be compromised for the participants of the study, but are available from the corresponding author on reasonable request.

## Abbreviations

BC: Breast Cancer
BMI: Body Mass Index
CI: Confidence Interval
DBCG: Danish Breast Cancer Group
ER: Estrogen Receptor
FISH: Fluorescence In Situ Hybridization
HbA_1c_: Glycosylated Hemoglobin
HbA_1c_-Q1: HbA_1c_ Quartile 1
HER2: Human Epidermal Growth Factor Receptor 2
HR: Hazard Ratio

## References

[1] Sung H, Ferlay J, Siegel RL, Laversanne M, Soerjomataram I, Jemal A, et al. Global cancer statistics 2020: GLOBOCAN estimates of incidence and mortality worldwide for 36 cancers in 185 countries. CA Cancer J Clin. 2021;71(3):209–49. 33538338 10.3322/caac.21660

[2] International Diabetes Federation. Facts & figures [Internet]. 2024 [cited 2024 Apr 15]. Available from: https://idf.org/about-diabetes/diabetes-facts-figures/

[3] World Health Organization. Obesity and overweight [Internet]. 2024 [cited 2024 Jan 9]. Available from: https://www.who.int/news-room/fact-sheets/detail/obesity-and-overweight

[4] Ward ZJ, Bleich SN, Cradock AL, Barrett JL, Giles CM, Flax C, et al. Projected U.S. State-Level prevalence of adult obesity and severe obesity. N Engl J Med. 2019;381(25):2440–50. 31851800 10.1056/NEJMsa1909301

[5] Xiong F, Dai Q, Zhang S, Bent S, Tahir P, Van Blarigan EL, et al. Diabetes and incidence of breast cancer and its molecular subtypes: A systematic review and meta-analysis. Diabetes Metab Res Rev. 2024;40(1):e3709. 37545374 10.1002/dmrr.3709

[6] National Cancer Institute. Obesity and Cancer [Internet]. 2024 [cited 2024 Mar 18]. Available from: https://www.cancer.gov/about-cancer/causes-prevention/risk/obesity/obesity-fact-sheet#r23

[7] Harborg S, Kjærgaard KA, Thomsen RW, Borgquist S, Cronin-Fenton D, Hjorth CF. New horizons: epidemiology of obesity, diabetes mellitus, and cancer prognosis. J Clin Endocrinol Metab. 2024;109(4):924–35. 37552777 10.1210/clinem/dgad450

[8] Kang C, LeRoith D, Gallagher EJ. Diabetes, obesity, and breast cancer. Endocrinology. 2018;159(11):3801–12. 30215698 10.1210/en.2018-00574 PMC6202853

[9] Xu F, Wang YF, Lu L, Liang Y, Wang Z, Hong X, et al. Comparison of anthropometric indices of obesity in predicting subsequent risk of hyperglycemia among Chinese men and women in Mainland China. Asia Pac J Clin Nutr. 2010;19(4):586–93. 21147722

[10] Tsilidis KK, Kasimis JC, Lopez DS, Ntzani EE, Ioannidis JPA. Type 2 diabetes and cancer: umbrella review of meta-analyses of observational studies. BMJ. 2015;350:g7607. 25555821 10.1136/bmj.g7607

[11] Ling S, Sweeting M, Zaccardi F, Adlam D, Kadam UT. Glycosylated haemoglobin and prognosis in 10,536 people with cancer and pre-existing diabetes: a meta-analysis with dose-response analysis. BMC Cancer. 2022;22(1):1048. 36203139 10.1186/s12885-022-10144-y PMC9535893

[12] Li W, Zhang X, Sang H, Zhou Y, Shang C, Wang Y, et al. Effects of hyperglycemia on the progression of tumor diseases. J Exp Clin Cancer Res. 2019;38(1):327. 31337431 10.1186/s13046-019-1309-6 PMC6651927

[13] Giugliano D, Ceriello A, Esposito K. Glucose metabolism and hyperglycemia. Am J Clin Nutr. 2008;87(1):S217–22. 10.1093/ajcn/87.1.217S 18175761

[14] Erickson K, Patterson RE, Flatt SW, Natarajan L, Parker BA, Heath DD, et al. Clinically defined type 2 diabetes mellitus and prognosis in early-stage breast cancer. J Clin Oncol. 2011;29(1):54–60. 21115861 10.1200/JCO.2010.29.3183 PMC3055860

[15] Chang YL, Sheu WHH, Lin SY, Liou WS. Good glycaemic control is associated with a better prognosis in breast cancer patients with type 2 diabetes mellitus. Clin Exp Med. 2018;18(3):383–90. 29572669 10.1007/s10238-018-0497-2

[16] Yoo TK, Lee MY, Lee SA, Cheong ES, Seo MH, Sung KC. Association of glycosylated hemoglobin level and Cancer-Related mortality in patients without diabetes. J Clin Med. 2022;11:19. 10.3390/jcm11195933 PMC9570990 36233800

[17] Tobe A, Horimoto Y, Kobayashi K, Kamisada N, Hirano M. Impact of diabetes on patient outcomes in breast cancer patients. Breast Care (Basel). 2022;17(5):480–5. 36684403 10.1159/000524513 PMC9851070

[18] Laurberg T, Witte DR, Gudbjörnsdottir S, Eliasson B, Bjerg L. Diabetes-related risk factors and survival among individuals with type 2 diabetes and breast, lung, colorectal, or prostate cancer. Sci Rep. 2024;14(1):10956. 38740921 10.1038/s41598-024-61563-9 PMC11091071

[19] Boursi B, Giantonio BJ, Lewis JD, Haynes K, Mamtani R, Yang YX. Serum glucose and hemoglobin A1C levels at cancer diagnosis and disease outcome. Eur J Cancer. 2016;59:90–8. 27017290 10.1016/j.ejca.2016.02.018 PMC4851868

[20] Jousheghany F, Phelps J, Crook T, Hakkak R. Relationship between level of HbA1C and breast cancer. BBA Clin. 2016;6:45–8. 27957429 10.1016/j.bbacli.2016.04.005 PMC5144103

[21] Joshu CE, Prizment AE, Dluzniewski PJ, Menke A, Folsom AR, Coresh J, et al. Glycated hemoglobin and cancer incidence and mortality in the atherosclerosis in communities (ARIC) study, 1990–2006. Int J Cancer. 2012;131(7):1667–77. 22161730 10.1002/ijc.27394 PMC3906204

[22] Sacks DB, Arnold M, Bakris GL, Bruns DE, Horvath AR, Lernmark Å, et al. Guidelines and recommendations for laboratory analysis in the diagnosis and management of diabetes mellitus. Diabetes Care. 2023;46(10):e151–99. 37471273 10.2337/dci23-0036 PMC10516260

[23] Holm JB, Baggesen E, Cronin-Fenton D, Frystyk J, Bruun JM, Christiansen P, et al. Circulating C-reactive protein levels as a prognostic biomarker in breast cancer across body mass index groups. Sci Rep. 2024;14(1):14486. 38914635 10.1038/s41598-024-64428-3 PMC11196728

[24] Møller S, Jensen MB, Ejlertsen B, Bjerre KD, Larsen M, Hansen HB, et al. The clinical database and the treatment guidelines of the Danish breast cancer cooperative group (DBCG); its 30-years experience and future promise. Acta Oncol. 2008;47(4):506–24. 18465317 10.1080/02841860802059259

[25] Jørgensen ME, Kristensen JK, Reventlov Husted G, Cerqueira C, Rossing P. The Danish adult diabetes registry. Clin Epidemiol. 2016;8:429–34. 27843339 10.2147/CLEP.S99518 PMC5098513

[26] Antonsen K, Rosenstock CV, Lundstrøm LH. The Danish anaesthesia database. Clin Epidemiol. 2016;8:435–8. 27843340 10.2147/CLEP.S99517 PMC5098505

[27] Schmidt M, Pedersen L, Sørensen HT. The Danish civil registration system as a tool in epidemiology. Eur J Epidemiol. 2014;29(8):541–9. 24965263 10.1007/s10654-014-9930-3

[28] Sundhedsdatastyrelsen. National health registers [Internet]. [cited 2025 Feb 10]. Available from: https://english.sundhedsdatastyrelsen.dk/health-data-and-registers/national-health-registers

[29] Regionernes Kliniske Kvalitetsudviklingsprogram. In English [Internet]. 2022 [cited 2024 Apr 11]. Available from: https://www.rkkp.dk/in-english/

[30] Bio- and Genome Bank Denmark. Bio- and Genome Bank Denmark [Internet]. 2022 [cited 2024 Apr 11]. Available from: https://www.regioner.dk/rbgben

[31] SEBIA. CAPILLARYS 3 TERA TLA [Internet]. 2024 [cited 2024 Apr 9]. Available from: https://www.sebia.com/instruments/capillarys-3-tera-tla/

[32] Hanas R, John G. 2010 Consensus statement on the worldwide standardization of the hemoglobin A1C measurement. Diabetes Care. 2010;33(8):1903–4. 20519665 10.2337/dc10-0953 PMC2909083

[33] Vistisen D, Witte DR, Brunner EJ, Kivimäki M, Tabák A, Jørgensen ME, et al. Risk of cardiovascular disease and death in individuals with prediabetes defined by different criteria: the Whitehall II study. Diabetes Care. 2018;41(4):899–906. 29453200 10.2337/dc17-2530 PMC6463620

[34] Weir CB, Jan A. In: StatPearls, editor. BMI classification percentile and cut off points. Treasure Island (FL): StatPearls Publishing; 2023. 31082114

[35] Charlson ME, Pompei P, Ales KL, MacKenzie CR. A new method of classifying prognostic comorbidity in longitudinal studies: development and validation. J Chronic Dis. 1987;40(5):373–83. 3558716 10.1016/0021-9681(87)90171-8

[36] Giuliano AE, Connolly JL, Edge SB, Mittendorf EA, Rugo HS, Solin LJ, et al. Breast cancer-Major changes in the American joint committee on cancer eighth edition cancer staging manual. CA Cancer J Clin. 2017;67(4):290–303. 28294295 10.3322/caac.21393

[37] Ahn S, Woo JW, Lee K, Park SY. HER2 status in breast cancer: changes in guidelines and complicating factors for interpretation. J Pathol Transl Med. 2020;54(1):34–44. 31693827 10.4132/jptm.2019.11.03 PMC6986968

[38] Sinn HP, Kreipe H. A Brief Overview of the WHO Classification of Breast Tumors, 4th Edition, Focusing on Issues and Updates from the 3rd Edition. Breast Care (Basel). 2013;8(2):149–54. 10.1159/000350774 PMC3683948 24415964

[39] Peduzzi P, Concato J, Feinstein AR, Holford TR. Importance of events per independent variable in proportional hazards regression analysis. II. Accuracy and precision of regression estimates. J Clin Epidemiol. 1995;48(12):1503–10. 8543964 10.1016/0895-4356(95)00048-8

[40] Viedma-Rodríguez R, Martínez-Hernández MG, Flores-López LA, Baiza-Gutman LA. Epsilon-aminocaproic acid prevents high glucose and insulin induced-invasiveness in MDA-MB-231 breast cancer cells, modulating the plasminogen activator system. Mol Cell Biochem. 2018;437(1–2):65–80. 28612231 10.1007/s11010-017-3096-8

[41] Flores-López LA, Martínez-Hernández MG, Viedma-Rodríguez R, Díaz-Flores M, Baiza-Gutman LA. High glucose and insulin enhance uPA expression, ROS formation and invasiveness in breast cancer-derived cells. Cell Oncol (Dordr). 2016;39(4):365–78. 27106722 10.1007/s13402-016-0282-8 PMC13001858

[42] Brabletz T, Kalluri R, Nieto MA, Weinberg RA. EMT in cancer. Nat Rev Cancer. 2018;18(2):128–34. 29326430 10.1038/nrc.2017.118

[43] James DE, Stöckli J, Birnbaum MJ. The aetiology and molecular landscape of insulin resistance. Nat Rev Mol Cell Biol. 2021;22(11):751–71. 34285405 10.1038/s41580-021-00390-6

[44] Donath MY, Shoelson SE. Type 2 diabetes as an inflammatory disease. Nat Rev Immunol. 2011;11(2):98–107. 21233852 10.1038/nri2925

[45] Goodwin PJ, Ennis M, Pritchard KI, Trudeau ME, Koo J, Madarnas Y, et al. Fasting insulin and outcome in early-stage breast cancer: results of a prospective cohort study. J Clin Oncol. 2002;20(1):42–51. 11773152 10.1200/JCO.2002.20.1.42

[46] Pierce BL, Ballard-Barbash R, Bernstein L, Baumgartner RN, Neuhouser ML, Wener MH, et al. Elevated biomarkers of inflammation are associated with reduced survival among breast cancer patients. J Clin Oncol. 2009;27(21):3437–44. 19470939 10.1200/JCO.2008.18.9068 PMC2717751

[47] Danish Breast Cancer Group. Rapporter [Internet]. [cited 2025 Feb 27]. Available from: https://dbcg.dk/kvalitetsdatabasen/rapporter

[48] Danske Regioner. Årsrapport Dansk Voksen Diabetes Database og Dansk Register for Børne - og Ungdomsdiabetes 2022 (in Danish) [Internet]. [cited 2024 Apr 15]. Available from: https://www.sundhed.dk/sundhedsfaglig/kvalitet/kliniske-kvalitetsdatabaser/kroniske-sygdomme/diabetes/

[49] Selvin E, Coresh J, Zhu H, Folsom A, Steffes MW. Measurement of HbA1c from stored whole blood samples in the atherosclerosis risk in communities study. J Diabetes. 2010;2(2):118–24. 20923494 10.1111/j.1753-0407.2010.00070.x PMC2991637

[50] Selvin E, Coresh J, Jordahl J, Boland L, Steffes MW. Stability of haemoglobin A1c (HbA1c) measurements from frozen whole blood samples stored for over a decade. Diabet Med. 2005;22(12):1726–30. 16401319 10.1111/j.1464-5491.2005.01705.x

