# Supplementary material for: HbA1c levels and breast cancer prognosis in women without diabetes
Source: BMC Cancer. 2025 Apr 28;25:790. doi: 10.1186/s12885-025-14121-z (PMC12036245; doi:10.1186/s12885-025-14121-z)
Supplement: Supplementary file 1 — Supplementary Material 1 [file 12885_2025_14121_MOESM1_ESM.docx]

**Supplementary materials**

**Supplementary Table 1:** Characteristics of 2336 women with incident stage I-III breast cancer with complete data

**Supplementary Table 2:** Outcome estimates according to HbA_1c_ quartiles and log2(HbA_1c_) with HbA_1c_ levels below 48 mmol/mol

**Supplementary Table 3:** Outcome estimates according to the International Expert Committee and American Diabetes Association HbA_1c_ cut-offs for diabetes

**Supplementary Table 4:** Outcome estimates according to HbA_1c_ quartiles and log2(HbA_1c_) stratified by estrogen receptor status

**Supplementary Table 5:** Outcome estimates according to HbA_1c_ quartiles and log2(HbA_1c_) across body mass index groups

**Supplementary Figure 1:** Directed acyclic graph including all descriptive characteristics with new breast cancer event and death as outcomes

**Supplementary Figure 2:** Cumulative new breast cancer event incidences according to HbA_1c_ quartiles across body mass index groups

**Supplementary Figure 3:** Cumulative all-cause mortality incidences according to HbA_1c_ quartiles across body mass index groups

**Supplementary Table 1. Characteristics of 2336 women with incident stage I-III breast cancer with complete data.**

| **Characteristics** | **Total**  N=2336 | **HbA_1c_-Q1**  21-33 mmol/mol N=606 | **HbA_1c_-Q2** 34-36 mmol/mol  N=734 | **HbA_1c_-Q3** 37-38 mmol/mol  N=455 | **HbA_1c_-Q4** $\geq$39 mmol/mol  N=541 |
| --- | --- | --- | --- | --- | --- |
| **Age, median** **(IQR)** | 62 (52-69) | 52 (47-64) | 60 (52-68) | 64 (57-70) | 66 (59-72) |
| **Age (years), categories** |  |  |  |  |  |
| < 50 | 414 (17.7%) | 214 (35.3%) | 127 (17.3%) | 42 (9.2%) | 31 (5.7%) |
| 50-59 | 608 (26.0%) | 172 (28.4%) | 218 (29.7%) | 105 (23.1%) | 113 (20.9%) |
| 60-69 | 797 (34.1%) | 146 (24.1%) | 251 (34.2%) | 190 (41.8%) | 210 (38.8%) |
| $\geq$ 70 | 517 (22.1%) | 74 (12.2%) | 138 (18.8%) | 118 (25.9%) | 187 (34.6%) |
| **Body Mass Index (kg/m^2^), median (IQR)** | 24.77 (22.23-28.17) | 23.59 (21.63-26.03) | 24.34 (22.04-27.58) | 25.01 (22.15-28.28) | 27.10 (24.11-31.25) |
| **Body Mass Index, categories (kg/m^2^)** |  |  |  |  |  |
| Underweight < 18.5 | 55 (2.4%) | 13 (2.1%) | 19 (2.6%) | 14 (3.1%) | 9 (1.7%) |
| Normal weight 18.5 $\leq$ to < 25 | 1157 (49.5%) | 383 (63.2%) | 389 (53.0%) | 209 (45.9%) | 176 (32.5%) |
| Overweight 25 ≤ to < 30 | 729 (31.2%) | 160 (26.4%) | 220 (30.0%) | 161 (35.4%) | 188 (34.8%) |
| Obesity ≥ 30 | 395 (16.9%) | 50 (8.3%) | 106 (14.4%) | 71 (15.6%) | 168 (31.1%) |
| **Menopausal status** |  |  |  |  |  |
| Premenopausal | 562 (24.1%) | 263 (43.4%) | 188 (25.6%) | 64 (14.1%) | 47 (8.7%) |
| Postmenopausal | 1774 (75.9%) | 343 (56.6%) | 546 (74.4%) | 391 (85.9%) | 494 (91.3%) |
| **Charlson Comorbidity Index** |  |  |  |  |  |
| 0 | 295 (12.6%) | 97 (16.0%) | 84 (11.4%) | 57 (12.5%) | 57 (10.5%) |
| 1-2 (mild) | 1724 (73.8%) | 441 (72.8%) | 568 (77.4%) | 346 (76.0%) | 369 (68.2%) |
| $\geq$3 (moderate/severe) | 317 (13.6%) | 68 (11.2%) | 82 (11.2%) | 52 (11.4%) | 115 (21.3%) |
| **Tumor size** |  |  |  |  |  |
| 0-20 mm | 1657 (70.9%) | 443 (73.1%) | 536 (73.0%) | 326 (71.6%) | 352 (65.1%) |
| 21-50 mm | 632 (27.1%) | 152 (25.1%) | 182 (24.8%) | 123 (27.0%) | 175 (32.3%) |
| >50 mm | 47 (2.0%) | 11 (1.8%) | 16 (2.2%) | 6 (1.3%) | 14 (2.6%) |
| **Lymph node metastases** |  |  |  |  |  |
| 0 | 1447 (61.9%) | 366 (60.4%) | 452 (61.6%) | 284 (62.4%) | 345 (63.8%) |
| 1-3 | 663 (28.4%) | 184 (30.4%) | 210 (28.6%) | 127 (27.9%) | 142 (26.2%) |
| 4-9 | 159 (6.8%) | 40 (6.6%) | 51 (6.9%) | 33 (7.3%) | 35 (6.5%) |
| $\geq$10 | 67 (2.9%) | 16 (2.6%) | 21 (2.9%) | 11 (2.4%) | 19 (3.5%) |
| **Histological classification** |  |  |  |  |  |
| Ductal | 1778 (76.1%) | 447 (73.8%) | 556 (75.7%) | 354 (77.8%) | 421 (77.8%) |
| Lobular | 299 (12.8%) | 87 (14.4%) | 100 (13.6%) | 51 (11.2%) | 61 (11.3%) |
| Other^a^ | 259 (11.1%) | 72 (11.9%) | 78 (10.6%) | 50 (11.0%) | 59 (10.9%) |
| **Histological grade** |  |  |  |  |  |
| Not graded^b^ | 125 (5.4%) | 36 (5.9%) | 39 (5.3%) | 25 (5.5%) | 25 (4.6%) |
| Grade 1 | 555 (23.8%) | 144 (23.8%) | 180 (24.5%) | 105 (23.1%) | 126 (23.3%) |
| Grade 2 | 1091 (46.7%) | 271 (44.7%) | 334 (45.5%) | 224 (49.2%) | 262 (48.4%) |
| Grade 3 | 565 (24.2%) | 155 (25.6%) | 181 (24.7%) | 101 (22.2%) | 128 (23.7%) |
| **ER status (% positive cells)** |  |  |  |  |  |
| 0% (negative) | 230 (9.8%) | 81 (13.4%) | 61 (8.3%) | 43 (9.5%) | 45 (8.3%) |
| 1-100% (positive) | 2106 (90.2%) | 525 (86.6%) | 673 (91.7%) | 412 (90.5%) | 496 (91.7%) |
| **HER2 status** |  |  |  |  |  |
| Negative | 2092 (89.6%) | 538 (88.8%) | 666 (90.7%) | 407 (89.5%) | 481 (88.9%) |
| Positive | 244 (10.4%) | 68 (11.2%) | 68 (9.3%) | 48 (10.5%) | 60 (11.1%) |
| **Final primary surgery**^c^ |  |  |  |  |  |
| Mastectomy | 765 (32.7%) | 204 (33.7%) | 232 (31.6%) | 139 (30.5%) | 190 (35.1%) |
| Lumpectomy | 1571 (67.3%) | 402 (66.3%) | 502 (68.4%) | 316 (69.5%) | 351 (64.9%) |
| **Adjuvant radiotherapy**^d^ |  |  |  |  |  |
| No | 454 (19.4%) | 113 (18.6%) | 139 (18.9%) | 84 (18.5%) | 118 (21.8%) |
| Yes | 1882 (80.6%) | 493 (81.4%) | 595 (81.1%) | 371 (81.5%) | 423 (78.2%) |
| **Endocrine therapy**^d^ |  |  |  |  |  |
| No | 444 (19.0%) | 137 (22.6%) | 122 (16.6%) | 88 (19.3%) | 97 (17.9%) |
| Yes | 1892 (81.0%) | 469 (77.4%) | 612 (83.4%) | 367 (80.7%) | 444 (82.1%) |
| **Anti-HER2 therapy**^d^ |  |  |  |  |  |
| No | 2092 (89.6%) | 538 (88.8%) | 666 (90.7%) | 407 (89.5%) | 481 (88.9%) |
| Yes | 244 (10.4%) | 68 (11.2%) | 68 (9.3%) | 48 (10.5%) | 60 (11.1%) |
| **Adjuvant chemotherapy**^d^ |  |  |  |  |  |
| No | 1143 (48.9%) | 225 (37.1%) | 357 (48.6%) | 248 (54.5%) | 313 (57.9%) |
| Yes | 1193 (51.1%) | 381 (62.9%) | 377 (51.4%) | 207 (45.5%) | 228 (42.1%) |
| *a: “Other” refers to patients without registration of either invasive ductal or lobular carcinoma.*  *b: In total, 125 patients’ tumors were not graded during the histological assessment, e.g. due to nonductal and nonlobular carcinomas were not graded for part of the study period or insufficient amount of tumor tissue for grading. We did not treat “Not graded” as a missing value in the multivariable models. c: Final primary surgery refers to the last breast surgery procedure for the primary breast cancer.*  *d: Intention-to-treat variables based on the Danish Breast Cancer Group protocol allocation.*  *Abbreviations: Q1, Quartile 1; IQR, Interquartile range; ER, Estrogen receptor; HER2, Human Epidermal Growth Factor Receptor 2.* | | | | | |

**Supplementary Table 2. Outcome estimates according to HbA_1c_ quartiles and log2(HbA_1c_) with HbA_1c_ levels below 48 mmol/mol**

|  | Person-years | Number of events | Incidence rate per 1000 person-years (95% CI) | Crude hazard ratio (95% CI) (N=2469) | Model 1: Hazard ratio adjusted for confounders based on directed acyclic graph (95% CI)^a^ (N=2404) | Model 2: Adjusted hazard ratio (95% CI)^b^ (N=2294) |
| --- | --- | --- | --- | --- | --- | --- |
| **New breast cancer event** | | | | | | |
| Q1 (21-33 mmol/mol) (N=649) | 3791 | 49 | 12.93 (9.77-17.10) | 1 [Reference] | 1 [Reference] | 1 [Reference] |
| Q2 (34-36 mmol/mol) (N=794) | 4547 | 64 | 14.08 (11.02-17.98) | 1.09 (0.75-1.58) | 1.10 (0.75-1.61) | 1.25 (0.84-1.88) |
| Q3 (37-38 mmol/mol) (N=485) | 2593 | 46 | 17.74 (13.29-23.68) | 1.37 (0.92-2.05) | 1.38 (0.90-2.11) | 1.40 (0.89-2.19) |
| Q4 (39-47 mmol/mol) (N=541) | 2957 | 66 | 22.32 (17.54-28.41) | 1.72 (1.19-2.49) | 1.75 (1.16-2.64) | 1.93 (1.24-3.00) |
| Total (N=2469) | 13888 | 225^c^ |  |  |  |  |
| Per HbA_1c_ log2 increase |  |  |  | 3.60 (1.48-8.77) | 3.48 (1.28-9.45) | 3.70 (1.27-10.77) |
| **Distant recurrence** | | | | | | |
| Q1 | 3791 | 27 | 7.12 (4.88-10.39) | 1 [Reference] | 1 [Reference] | NA |
| Q2 | 4547 | 38 | 8.36 (6.08-11.49) | 1.18 (0.72-1.93) | 1.25 (0.76-2.07) | NA |
| Q3 | 2593 | 26 | 10.03 (6.83-14.73) | 1.42 (0.83-2.44) | 1.57 (0.89-2.77) | NA |
| Q4 | 2957 | 42 | 14.20 (10.50-19.22) | 2.01 (1.24-3.26) | 2.17 (1.27-3.72) | NA |
| Total | 13888 | 133^d^ |  |  |  |  |
| Per HbA_1c_ log2 increase |  |  |  | 4.82 (1.51-15.34) | 5.19 (1.41-19.10) | NA |
| **All-cause mortality** | | | | | | |
| Q1 | 3987 | 57 | 14.30 (11.03-18.53) | 1 [Reference] | 1 [Reference] | 1 [Reference] |
| Q2 | 4787 | 65 | 13.58 (10.65-17.31) | 0.95 (0.67-1.36) | 0.75 (0.52-1.07) | 0.81 (0.55-1.20) |
| Q3 | 2723 | 54 | 19.83 (15.19-25.89) | 1.41 (0.97-2.05) | 0.82 (0.55-1.21) | 0.79 (0.52-1.20) |
| Q4 | 3154 | 83 | 26.32 (21.22-32.63) | 1.86 (1.32-2.60) | 1.07 (0.74-1.54) | 1.13 (0.76-1.67) |
| Total | 14652 | 259 |  |  |  |  |
| Per HbA_1c_ log2 increase |  |  |  | 3.49 (1.53-7.98) | 0.81 (0.33-1.96) | 0.76 (0.29-1.97) |
| *a: Adjusted for age, menopausal status, comorbidities, and body mass index*  *b: Adjusted for age, menopausal status, comorbidities, body mass index, estrogen receptor status, HER2 receptor status, histological grade, tumor size, lymph node metastases, histological classification, surgery, radiotherapy, and systemic treatment (endocrine therapy, chemotherapy, and anti-HER2 therapy)*  *c: 190 breast cancer recurrences, 35 contralateral breast cancers*  *d: 81 visceral metastases and 52 bone metastases*  *Abbreviations: Q1, HbA_1c_ Quartile 1* | | | | | | |

**Supplementary Table 3. Outcome estimates according to the International Expert Committee and American Diabetes Association HbA_1c_ cut-offs for diabetes**(1)**.**

***Supplementary Table 3a. International Expert Committee***

| HbA_1c_ | Person-years | Number of events | Incidence rate per 1000 person-years (95% CI) | Crude hazard ratio (95% CI) (N=2514) | Model 1: Hazard ratio adjusted for confounders based on directed acyclic graph (95% CI)^a^ (N=2448) | Model 2: Adjusted hazard ratio (95% CI)^b^ (N=2336) |
| --- | --- | --- | --- | --- | --- | --- |
| **New breast cancer event** | | | | | | |
| $\leq$41 mmol/mol (N=2290) | 12986 | 202 | 15.55 (13.55-17.85) | 1 [Reference] | 1 [Reference] | 1 [Reference] |
| 42-47 mmol/mol (N=179) | 902 | 23 | 25.50 (16.95-38.38) | 1.62 (1.05-2.50) | 1.47 (0.92-2.34) | 1.50 (0.91-2.47) |
| $\geq$48 mmol/mol (N=45) | 238 | 5 | 21.03 (8.75-50.52) | 1.36 (0.56-3.31) | 1.07 (0.40-2.92) | 0.88 (0.32-2.43) |
| Total (N=2514) | 14126 | 230^c^ |  |  |  |  |
| **Distant recurrence** | | | | | | |
| $\leq$41 mmol/mol | 12986 | 119 | 9.16 (7.66-10.97) | 1 [Reference] | 1 [Reference] | NA |
| 42-47 mmol/mol | 902 | 14 | 15.52 (9.19-26.21) | 1.69 (0.97-2.94) | 1.48 (0.80-2.73) | NA |
| $\geq$48 mmol/mol | 238 | 4 | 16.82 (6.31-44.82) | 1.85 (0.68-5.02) | 1.35 (0.42-4.31) | NA |
| Total | 14126 | 137^d^ |  |  |  |  |
| **All-cause mortality** | | | | | | |
| $\leq$41 mmol/mol | 13690 | 230 | 16.80 (14.76-19.12) | 1 [Reference] | 1 [Reference] | 1 [Reference] |
| 42-47 mmol/mol | 961 | 29 | 30.17 (20.96-43.41) | 1.84 (1.25-2.70) | 1.20 (0.79-1.81) | 1.26 (0.81-1.97) |
| $\geq$48 mmol/mol | 261 | 8 | 30.66 (15.33-61.30) | 1.82 (0.90-3.68) | 1.14 (0.53-2.44) | 0.92 (0.40-2.12) |
| Total | 14913 | 267 |  |  |  |  |
| *a: Adjusted for age, menopausal status, comorbidities, and body mass index*  *b: Adjusted for age, menopausal status, comorbidities, body mass index, estrogen receptor status, HER2 receptor status, histological grade, tumor size, lymph node metastases, histological classification, surgery, radiotherapy, and systemic treatment (endocrine therapy, chemotherapy, and anti-HER2 therapy)*  *c: 195 breast cancer recurrences, 35 contralateral breast cancers*  *d: 83 visceral metastases and 54 bone metastases* | | | | | | |

***Supplementary Table 3b. American Diabetes Association***

| HbA_1c_ | Person-years | Number of events | Incidence rate per 1000 person-years (95% CI) | Crude hazard ratio (95% CI) (N=2514) | Model 1: Hazard ratio adjusted for confounders based on directed acyclic graph (95% CI)^a^ (N=2448) | Model 2: Adjusted hazard ratio (95% CI)^b^ (N=2336) |
| --- | --- | --- | --- | --- | --- | --- |
| **New breast cancer event** | | | | | | |
| $\leq$38 mmol/mol (N=1928) | 10931 | 159 | 14.55 (12.45-16.99) | 1 [Reference] | 1 [Reference] | 1 [Reference] |
| 39-47 mmol/mol (N=541) | 2957 | 66 | 22.32 (17.54-28.41) | 1.53 (1.15-2.04) | 1.50 (1.10-2.04) | 1.53 (1.11-2.13) |
| $\geq$48 mmol/mol (N=45) | 238 | 5 | 21.03 (8.75-50.52) | 1.46 (0.60-3.55) | 1.19 (0.43-3.24) | 0.99 (0.36-2.73) |
| Total (N=2514) | 14126 | 230^c^ |  |  |  |  |
| **Distant recurrence** | | | | | | |
| $\leq$38 mmol/mol | 10931 | 91 | 8.32 (6.78-10.22) | 1 [Reference] | 1 [Reference] | NA |
| 39-47 mmol/mol | 2957 | 42 | 14.20 (10.50-19.22) | 1.71 (1.19-2.47) | 1.68 (1.12-2.50) | NA |
| $\geq$48 mmol/mol | 238 | 4 | 16.82 (6.31-44.82) | 2.04 (0.75-5.55) | 1.56 (0.48-5.01) | NA |
| Total | 14126 | 137^d^ |  |  |  |  |
| **All-cause mortality** | | | | | | |
| $\leq$38 mmol/mol | 11498 | 176 | 15.31 (13.20-17.74) | 1 [Reference] | 1 [Reference] | 1 [Reference] |
| 39-47 mmol/mol | 3154 | 83 | 26.32 (21.22-32.63) | 1.72 (1.33-2.24) | 1.29 (0.98-1.70) | 1.32 (0.99-1.78) |
| $\geq$48 mmol/mol | 261 | 8 | 30.66 (15.33-61.30) | 1.99 (0.98-4.05) | 1.22 (0.57-2.64) | 1.00 (0.43-2.33) |
| Total | 14913 | 267 |  |  |  |  |
| *a: Adjusted for age, menopausal status, comorbidities, and body mass index*  *b: Adjusted for age, menopausal status, comorbidities, body mass index, estrogen receptor status, HER2 receptor status, histological grade, tumor size, lymph node metastases, histological classification, surgery, radiotherapy, and systemic treatment (endocrine therapy, chemotherapy, and anti-HER2 therapy)*  *c: 195 breast cancer recurrences, 35 contralateral breast cancer*  *d: 83 visceral metastases and 54 bone metastases* | | | | | | |

**Supplementary Table 4. Outcome estimates according to HbA_1c_ quartiles and log2(HbA_1c_) stratified by estrogen receptor status**

|  | Person-years | Number of events | Incidence rate per 1000 person-years (95% CI) | Crude hazard ratio (95% CI) | Model 1: Hazard ratio adjusted for confounders based on directed acyclic graph (95% CI)^a^ |
| --- | --- | --- | --- | --- | --- |
| **Estrogen receptor positive** | | | | | |
| **New breast cancer event** |  |  |  | N=2249 | N=2195 |
| Q1 (21-33 mmol/mol) N=559 | 3285 | 35 | 10.65 (7.65-14.84) | 1 [Reference] | 1 [Reference] |
| Q2 (34-36 mmol/mol) N=722 | 4135 | 54 | 13.06 (10.00-17.05) | 1.23 (0.80-1.88) | 1.18 (0.77-1.83) |
| Q3 (37-38 mmol/mol) N=438 | 2307 | 36 | 15.60 (11.26-21.63) | 1.47 (0.92-2.34) | 1.41 (0.87-2.29) |
| Q4 ($\geq$39 mmol/mol) N=530 | 2930 | 55 | 18.77 (14.41-24.45) | 1.76 (1.15-2.70) | 1.66 (1.04-2.65) |
| Total (N=2249) | 12658 | 180^b^ |  |  |  |
| Per HbA_1c_ log2 increase |  |  |  | 2.35 (1.14-4.88) | 1.96 (0.83-4.62) |
| **All-cause mortality** |  |  |  |  |  |
| Q1 | 3458 | 38 | 10.99 (8.00-15.10) | 1 [Reference] | 1 [Reference] |
| Q2 | 4366 | 56 | 12.83 (9.87-16.67) | 1.17 (0.78-1.77) | 0.87 (0.57-1.33) |
| Q3 | 2419 | 44 | 18.19 (13.54-24.44) | 1.71 (1.10-2.63) | 0.95 (0.61-1.49) |
| Q4 | 3109 | 75 | 24.13 (19.24-30.25) | 2.22 (1.50-3.28) | 1.17 (0.77-1.78) |
| Total | 13352 | 213 |  |  |  |
| Per HbA_1c_ log2 increase |  |  |  | 3.81 (2.13-6.81) | 1.49 (0.67-3.30) |
| **Estrogen receptor negative** | | | | | |
| **New breast cancer event** |  |  |  | N=252 | N=241 |
| Q1 (N=89) | 505 | 13 | 25.77 (14.96-44.37) | 1 [Reference] | 1 [Reference] |
| Q2 (N=67) | 388 | 10 | 25.79 (13.88-47.94) | 1.06 (0.46-2.41) | 1.29 (0.55-3.05) |
| Q3 (N=45) | 278 | 10 | 35.92 (19.33-66.76) | 1.45 (0.64-3.32) | 1.41 (0.55-3.65) |
| Q4 (N=51) | 250 | 15 | 59.96 (36.15-99.46) | 2.31 (1.10-4.86) | 2.41 (1.01-5.74) |
| Total (N=252) | 1421 | 48^c^ |  |  |  |
| Per HbA_1c_ log2 increase |  |  |  | 3.79 (0.80-17.89) | 3.02 (0.51-18.05) |
| **All-cause mortality** |  |  |  |  |  |
| Q1 | 528 | 18 | 34.10 (21.48-54.12) | 1 [Reference] | 1 [Reference] |
| Q2 | 397 | 9 | 22.69 (11.80-43.60) | 0.69 (0.31-1.53) | 0.68 (0.30-1.56) |
| Q3 | 297 | 10 | 33.71 (18.14-62.66) | 1.02 (0.47-2.20) | 0.56 (0.24-1.34) |
| Q4 | 281 | 15 | 53.42 (32.20-88.60) | 1.57 (0.79-3.11) | 1.18 (0.54-2.62) |
| Total | 1502 | 52 |  |  |  |
| Per HbA_1c_ log2 increase |  |  |  | 0.80 (0.18-3.51) | 0.35 (0.06-1.86) |
| *a: Adjusted for age, menopausal status, body mass index, and comorbidities.*  *b: 154 breast cancer recurrences, 26 contralateral breast cancers*  *c: 39* *breast cancer recurrences, 9 contralateral breast cancers*  *Abbreviations: Q1, HbA_1c_ Quartile 1* | | | | | |

**Supplementary Table 5. Outcome estimates according to HbA_1c_ quartiles and log2(HbA_1c_) across body mass index groups**

|  | Person-years | Number of events | Incidence rate per 1000 person-years (95% CI) | Crude hazard ratio (95% CI) | Model 1: Hazard ratio adjusted for confounders based on directed acyclic graph (95% CI)^a^ |
| --- | --- | --- | --- | --- | --- |
| **Normal weight (18.5 ≤ BMI < 25 kg/m^2^)** | | | | | |
| **New breast cancer event** |  |  |  | N=1229 | N=1210 |
| Q1 (21-33 mmol/mol) N=406 | 2383 | 32 | 13.43 (9.49-18.99) | 1 [Reference] | 1 [Reference] |
| Q2 (34-35 mmol/mol) N=247 | 1415 | 14 | 9.90 (5.86-16.71) | 0.74 (0.39-1.38) | 0.83 (0.44-1.57) |
| Q3 (36-37 mmol/mol) N=309 | 1726 | 25 | 14.48 (9.79-21.43) | 1.08 (0.64-1.82) | 1.25 (0.72-2.18) |
| Q4 (38-61 mmol/mol) N=267 | 1429 | 34 | 23.79 (17.00-33.30) | 1.77 (1.09-2.87) | 2.14 (1.24-3.68) |
| Total (N=1229) | 6953 | 105^b^ |  |  |  |
| Per HbA_1c_ log2 increase |  |  |  | 2.34 (0.67-8.18) | 3.01 (0.79-11.55) |
| **All-cause mortality** |  |  |  |  |  |
| Q1 | 2479 | 32 | 12.91 (9.13-18.25) | 1 [Reference] | 1 [Reference] |
| Q2 | 1450 | 16 | 11.04 (6.76-18.01) | 0.86 (0.47-1.57) | 0.69 (0.37-1.26) |
| Q3 | 1806 | 29 | 16.06 (11.16-23.11) | 1.25 (0.76-2.06) | 0.74 (0.44-1.24) |
| Q4 | 1505 | 45 | 29.89 (22.32-40.03) | 2.33 (1.48-3.66) | 1.16 (0.72-1.89) |
| Total | 7240 | 122 |  |  |  |
| Per HbA_1c_ log2 increase |  |  |  | 6.84 (2.28-20.52) | 1.12 (0.36-3.49) |
| **Overweight (25 ≤ BMI < 30 kg/m^2^)** | | | | | |
| **New breast cancer event** |  |  |  | N=769 | N=764 |
| Q1 (25-34 mmol/mol) N=235 | 1339 | 22 | 16.43 (10.82-24.95) | 1 [Reference] | 1 [Reference] |
| Q2 (35-36 mmol/mol) N=159 | 940 | 14 | 14.89 (8.82-25.15)) | 0.90 (0.46-1.76) | 0.86 (0.44-1.69) |
| Q3 (37-39 mmol/mol) N=219 | 1186 | 29 | 24.46 (16.99-35.19) | 1.48 (0.85-2.57) | 1.38 (0.78-2.45) |
| Q4 (40-111 mmol/mol) N=156 | 850 | 16 | 18.83 (11.54-30.74) | 1.14 (0.60-2.18) | 1.05 (0.54-2.05) |
| Total (N=769) | 4315 | 81^c^ |  |  |  |
| Per HbA_1c_ log2 increase |  |  |  | 1.63 (0.55-4.83) | 1.49 (0.46-4.82) |
| **All-cause mortality** |  |  |  |  |  |
| Q1 | 1431 | 29 | 20.27 (14.08-29.16) | 1 [Reference] | 1 [Reference] |
| Q2 | 1011 | 9 | 8.90 (4.63-17.10) | 0.44 (0.21-0.92) | 0.40 (0.19-0.84) |
| Q3 | 1266 | 24 | 18.96 (12.71-28.28) | 0.95 (0.55-1.63) | 0.76 (0.43-1.32) |
| Q4 | 907 | 23 | 25.37 (16.86-38.18) | 1.26 (0.73-2.18) | 0.90 (0.51-1.59) |
| Total | 4615 | 85 |  |  |  |
| Per HbA_1c_ log2 increase |  |  |  | 1.94 (0.71-5.34) | 1.34 (0.42-4.26) |
| **Obesity (BMI** $\boldsymbol{\geq}$ **30 kg/m^2^)** | | | | | |
| **New breast cancer event** |  |  |  | N=416 | N=412 |
| Q1 (26-35 mmol/mol) N=114 | 683 | 5 | 7.32 (3.05-17.58) | 1 [Reference] | 1 [Reference] |
| Q2 (36-38 mmol/mol) N=126 | 658 | 9 | 13.68 (7.12-26.28) | 1.88 (0.63-5.62) | 1.74 (0.58-5.25) |
| Q3 (39-41 mmol/mol) N=96 | 547 | 8 | 14.62 (7.31-29.24) | 2.01 (0.66-6.14) | 1.87 (0.60-5.84) |
| Q4 (42-78 mmol/mol) N=80 | 399 | 11 | 27.59 (15.28-49.82) | 3.91 (1.35-11.27) | 3.07 (0.99-9.54) |
| Total (N=416) | 2287 | 33^d^ |  |  |  |
| Per HbA_1c_ log2 increase |  |  |  | 4.29 (1.07-17.17) | 3.26 (0.67-15.95) |
| **All-cause mortality** |  |  |  |  |  |
| Q1 | 702 | 9 | 12.82 (6.67-24.64) | 1 [Reference] | 1 [Reference] |
| Q2 | 679 | 9 | 13.25 (6.89-25.46) | 1.07 (0.43-2.71) | 0.75 (0.29-1.92) |
| Q3 | 582 | 13 | 22.32 (12.96-38.45) | 1.76 (0.75-4.11) | 1.18 (0.50-2.78) |
| Q4 | 450 | 9 | 20.00 (10.40-38.42) | 1.58 (0.63-3.98) | 0.64 (0.24-1.69) |
| Total | 2414 | 40 |  |  |  |
| Per HbA_1c_ log2 increase |  |  |  | 2.17 (0.57-8.23) | 0.59 (0.12-2.88) |
| *a: Adjusted for age, menopausal status, and comorbidities.*  *b: 84 breast cancer recurrences, 21 contralateral breast cancers*  *c: 72* *breast cancer recurrences, 9 contralateral breast cancers*  *d: 28 breast cancer recurrences, 5 contralateral breast cancers*  *Abbreviations: Q1, HbA_1c_ Quartile 1; BMI, Body Mass Index* | | | | | |

**Supplementary Figure 1. Directed acyclic graph including all descriptive characteristics with new breast cancer event and death as outcomes**

**
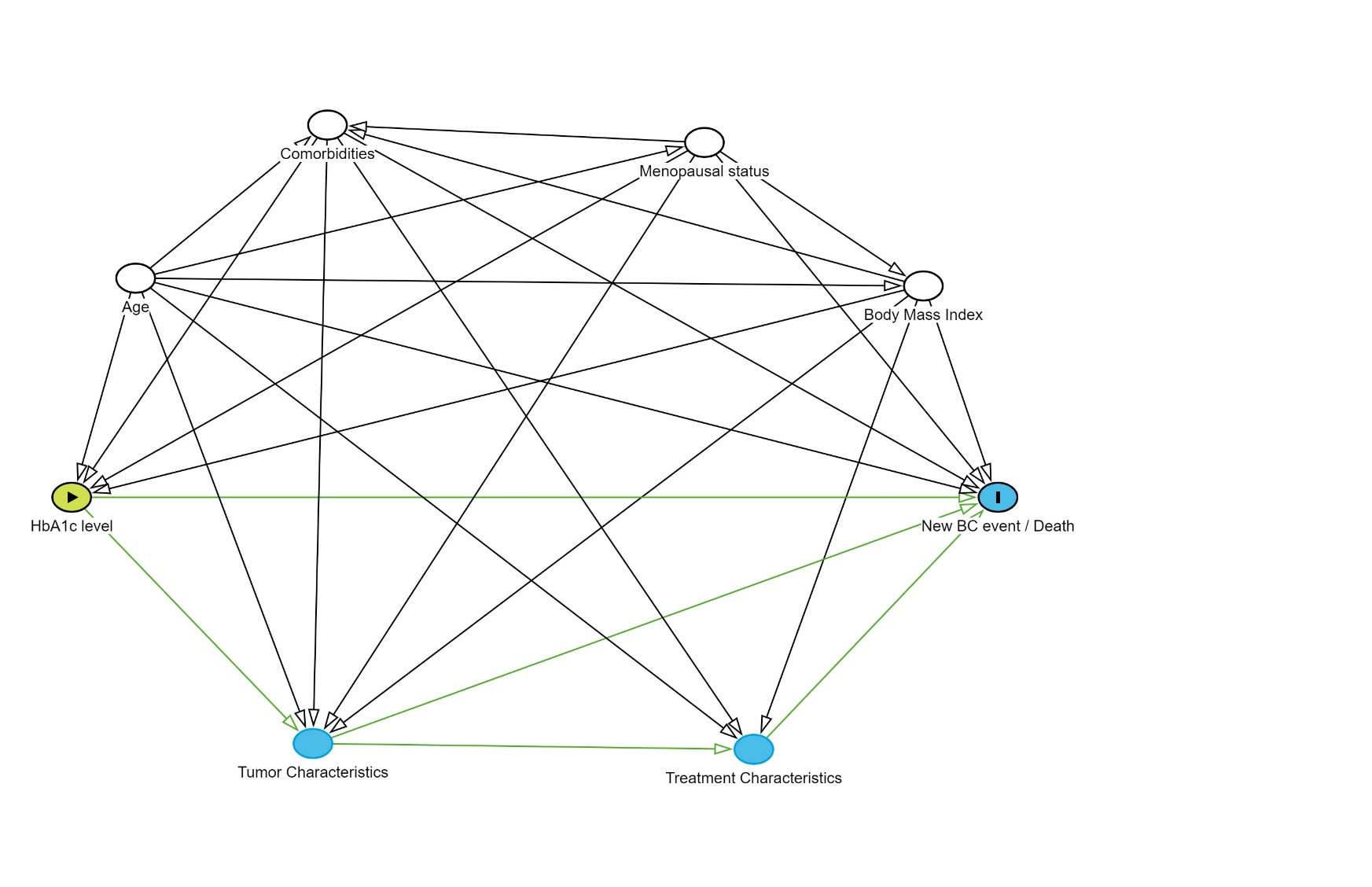
**

**Supplementary Figure 1 legend**: Tumor characteristics include tumor size, lymph node metastases, histological classification, histological grade, estrogen receptor status, and HER2 receptor status. Treatment characteristics include final primary surgery, adjuvant radiotherapy (intention-to-treat), adjuvant chemotherapy (intention-to-treat), adjuvant endocrine therapy (intention-to-treat), and adjuvant anti-HER2 therapy (intention-to-treat). Comorbidities include comorbidities in the Charlson Comorbidity Index(2). New breast cancer event includes breast cancer recurrence and contralateral breast cancer. The directed acyclic graph was created on the website <https://www.dagitty.net/dags.html>. Abbreviations: BC, Breast cancer.

**Supplementary Figure 2. Cumulative new breast cancer event incidences according to HbA_1c_ quartiles across body mass index groups**

***Supplementary Figure 2a***

**

**

***Supplementary Figure 2b***

**

**

***Supplementary Figure 2c***

******

******

**Supplementary Figure 2 legend:** Cumulative new breast cancer event (breast cancer recurrence or contralateral breast cancer) incidences according to HbA_1c_ quartiles across BMI groups (Aalen-Johansen estimator). Competing events: new primary cancer other than breast cancer and death.
2a: Cumulative new breast cancer event incidences across HbA_1c_ quartiles in patients with normal weight (18.5 ≤ BMI < 25 kg/m^2^).
2b: Cumulative new breast cancer event incidences across HbA_1c_ quartiles in patients with overweight (25 ≤ BMI < 30 kg/m^2^).
2c: Cumulative new breast cancer event incidences across HbA_1c_ quartiles in patients with obesity (BMI $\geq$ 30 kg/m^2^).
Abbreviations: BC, Breast cancer; BMI, Body Mass Index; HbA1c Q1, HbA_1c_ quartile 1.

**Supplementary Figure 3. Cumulative all-cause mortality incidences according to HbA_1c_ quartiles across body mass index groups**

***Supplementary Figure 3a***

***Supplementary Figure 3b***

***Supplementary Figure 3c***

**Supplementary Figure 3 legend:** Cumulative all-cause mortality incidences according to HbA_1c_ quartiles across BMI groups (Kaplan-Meier estimator).
3a: Cumulative all-cause mortality incidences across HbA_1c_ quartiles in patients with normal weight (18.5 ≤ BMI < 25 kg/m^2^).
3b: Cumulative all-cause mortality incidences across HbA_1c_ quartiles in patients with overweight (25 ≤ BMI < 30 kg/m^2^).
3c: Cumulative all-cause mortality incidences across HbA_1c_ quartiles in patients with obesity (BMI $\geq$ 30 kg/m^2^).
Abbreviations: BMI, Body Mass Index; HbA1c Q1, HbA_1c_ quartile 1.

**References**

1. Vistisen D, Witte DR, Brunner EJ, Kivimäki M, Tabák A, Jørgensen ME, et al. Risk of Cardiovascular Disease and Death in Individuals With Prediabetes Defined by Different Criteria: The Whitehall II Study. Diabetes Care. 2018 Apr;41(4):899–906.

2. Charlson ME, Pompei P, Ales KL, MacKenzie CR. A new method of classifying prognostic comorbidity in longitudinal studies: development and validation. J Chronic Dis. 1987;40(5):373–83.
